# The molecular evolution of genes previously associated with large sizes reveals possible pathways to cetacean gigantism

**DOI:** 10.1038/s41598-022-24529-3

**Published:** 2023-01-19

**Authors:** Felipe André Silva, Érica M. S. Souza, Elisa Ramos, Lucas Freitas, Mariana F. Nery

**Affiliations:** Laboratório de Genômica Evolutiva, Departamento de Genética, Evolução, Microbiologia e Imunologia, Instituto de Biologia, Universidade Estadual de Campinas-UNICAMP, 255, Monteiro Lobato, Cidade Universitária, IB, Bloco H, Campinas, SP 13083-862 Brazil

**Keywords:** Evolution, Molecular evolution

## Abstract

Cetaceans are a group of aquatic mammals with the largest body sizes among living animals, including giant representatives such as blue and fin whales. To understand the genetic bases of gigantism in cetaceans, we performed molecular evolutionary analyses on five genes (GHSR, IGF2, IGFBP2, IGFBP7, and EGF) from the growth hormone/insulin-like growth factor axis, and four genes (ZFAT, EGF, LCORL, and PLAG1) previously described as related to the size of species evolutionarily close to cetaceans, such as pigs, cows, and sheep. Our dataset comprised 19 species of cetaceans, seven of which are classified as giants because they exceed 10 m in length. Our results revealed signs of positive selection in genes from the growth hormone/insulin-like growth factor axis and also in those related to body increase in cetacean-related species. In addition, pseudogenization of the EGF gene was detected in the lineage of toothless cetaceans, Mysticeti. Our results suggest the action of positive selection on gigantism in genes that act both in body augmentation and in mitigating its consequences, such as cancer suppression when involved in processes such as division, migration, and cell development control.

## Introduction

Gigantism results from species evolving huge body sizes relative to their small-bodied ancestors. This phenomenon has been extensively studied because it affects critical life-history traits such as longevity, fecundity, and health^1–4^. However, it is still unclear how natural selection favors great body size from an evolutionary perspective. Gigantism may bring consequences such as an overall reduction in the genetic effective population size (N_e_) due to lower population densities^5^, lower reproductive output^6^, and the need to develop suppression mechanisms for diseases such as cancer, given the large number of cells needed to constitute a giant organism^7^. Despite this, several lineages of terrestrial and aquatic animals became giants throughout the history of life. Examples of these lineages can be found across the entire tree of life, such as tortoises^8^, sloths^9^, dinosaurs^10^, and aquatic animals such as the extinct *Jaekelopterus rhenaniae*—the largest arthropod ever found^11^—and the reptiles *Mosasaurus hoffmanni*^12^ and *Shonisaurus sikanniensis*^13^.

Aquatic and terrestrial habitats impose different selective pressures on body size, with aquatic organisms typically reaching larger proportions than their terrestrial relatives^14^. Several reasons have been proposed to explain this difference, for example, thermoregulation, abundant high-quality food in the aquatic environment, and a wider space available to explore new niches and specializations^15,16^.

Cetaceans (whales, porpoises, and dolphins) are aquatic mammals that evolved from small terrestrial ancestors around 50 million years ago during the Eocene^17^. The recolonization of the aquatic environment was followed by many morphological and physiological modifications, such as streamlined bodies, loss of body hair to reduce friction during swimming, reduced olfactory and gustatory systems, and hindlimb loss^18^. Currently, there are approximately 86 species of cetaceans, and these animals are divided into two groups: odontocetes (toothed whales) and mysticetes (whales with baleen that allow the filtration of food)^19^. One notable characteristic of cetaceans is the large size of several species. For example, the blue whale (*Balaenoptera musculus*) is the largest animal known to have existed, measuring 30 m long, and weighing more than 150 tons^20^. Also, the sperm whale (*Physeter catodon)* can reach up to 20 m and is the largest toothed animal living today. Several other cetaceans are known for their large bodies, such as the 25 m long fin whale (*Balaenoptera physalus*)^21^, 19 m long humpback whale (*Megaptera novaeangliae*)^22^, 17 m long bowhead whale (*Balaena mysticetus*)^23^, and 15 m long gray whale (*Eschrichtius robustus*)^24^. One of the main hypotheses to explain the large size of cetaceans relates to how they obtain food. It is argued that toothed cetaceans (i.e., odontocetes) developed large bodies due to the ability to dive and exploit prey from the seabed using a powerful biosonar, while in baleen whales (i.e., mysticetes) the evolution of gigantism is associated with the highly efficient exploitation of small prey^25,26^.

From a molecular point of view, body length is a complex character associated with many genes^27^. In mammals, research is mainly focused on domesticated species related to meat and milk production, due to their economic importance. In this context, many genes involved in body size growth have been described, such as the transcription factor LCORL (Ligand Dependent Nuclear Receptor Corepressor Like), which is responsible for size differences in sheep; NCAPG (Non-SMC Condensin I Complex Subunit G), PLAG1 (Pleomorphic Adenoma Gene 1), which acts in prenatal growth and is associated to body size of cattle; and ZFAT (Zinc Finger And AT-Hook Domain Containing) related to embryonic development and growth in human populations and horses^28–30^. Also, the growth hormone/insulin-like growth factor (GH-IGF) axis has been associated with growth rates, such GHSR (Growth Hormone Secretagogue Receptor), IGFBP2 (Insulin-Like Growth Factor Binding Protein 2), IGFBP7 (Insulin-Like Growth Factor Binding Protein 7), IGF2 (Insulin-Like Growth Factor 2), and EGF (Epidermal Growth Factor) genes^31^. Furthermore, since the somatotropic axis plays a central role in regulating growth, any locus expressing hormones, factors, or peptides within this system may reasonably represent a potential gene of significant importance in enhancing growth.

Accordingly, we aimed to investigate the molecular evolution of genes related to body size in cetaceans in a phylogenetic framework. We focused on five genes from the growth hormone/insulin-like growth factor (GH-IGF) axis, and four genes previously associated with increased body size in other cetartiodactyl species. Our main goal is to expand our understanding of the genetic basis of the morphological phenotypic variability of cetaceans.

## Results

Among the nine selected genes to perform the molecular evolution analyses, we found that in the EGF gene, stop codons resulted in the interruption of the reading frame only in the Mysticeti cetacean lineage. To our knowledge, this is the first time that EGF pseudogenization has been reported for mysticetes. As this work focuses on the coding regions, only the results for the other eight genes are described below.

### Selection analyses

#### Branch model

**Figure 1:**
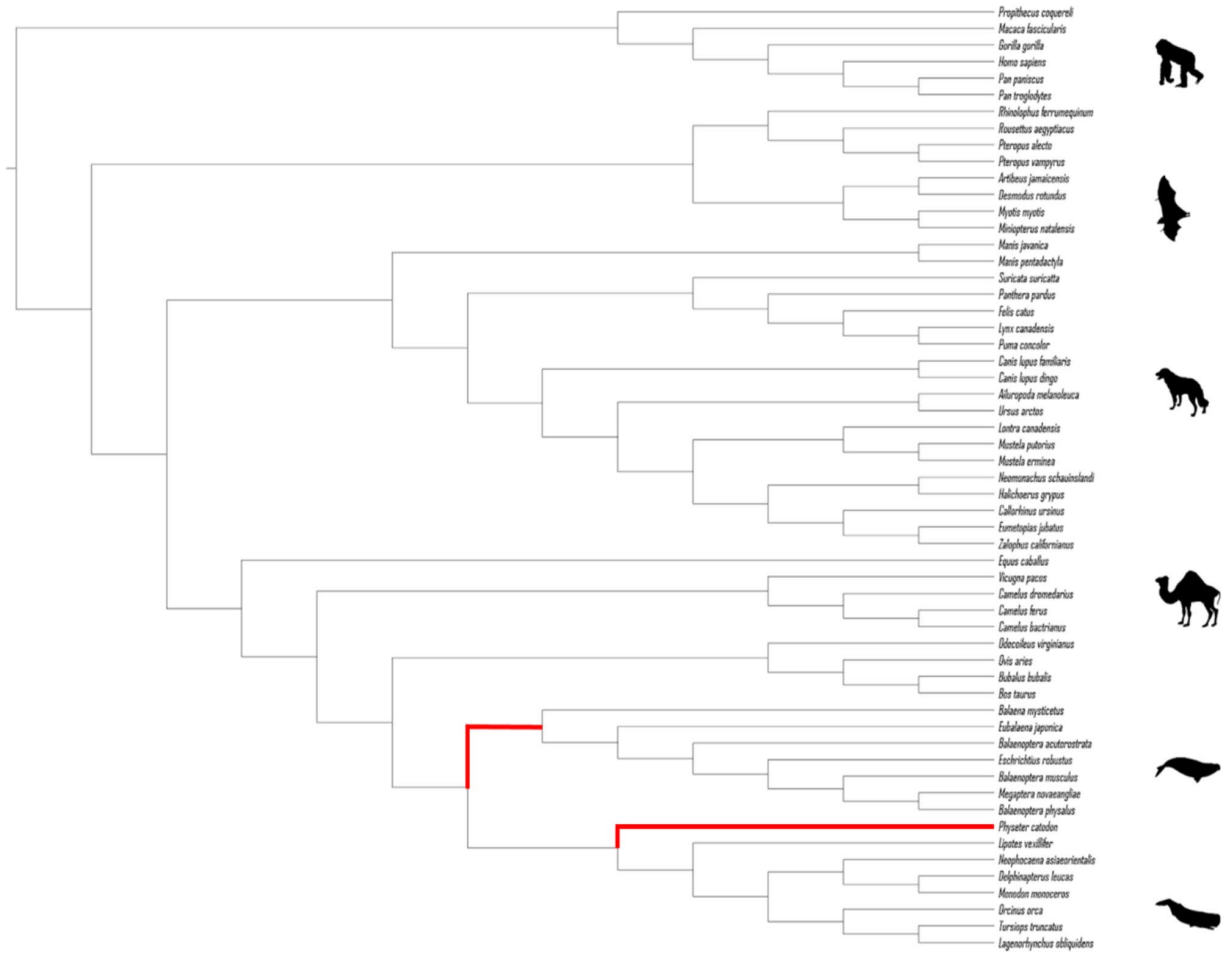
The species tree used in this study illustrates the group of giant cetaceans and the other mammals included in our dataset. In red, selection of ancestral branches that give rise to gigantism. Mammalian phylogeny is based on Beck and Baillie^86^, while phylogenetic relationships among cetaceans are based on McGowen et al.^87^.

Branch analyses were performed using species trees. First, we labeled the ancestral branch that led to the giant cetaceans, i.e., the stem mysticete lineage and the branch of the sperm whale (*Physeter catodon*), since this is the only giant species of odontocete group, exceeding 10 m in body size (Fig. 1). Then we compare the rates between giant and non-giant cetaceans, labeling all species with body size larger than 10 m as one group.

For the first labeled scheme, codeML free-ratio test fitted our data significantly better than the one-ratio model for all genes. However, the two-ratio model, used to estimate whether giant cetaceans have a different omega value compared to the other species, fitted better for PLAG1. In the second scheme, no statistically significant differences were found using codeML.

**Figure 2:**
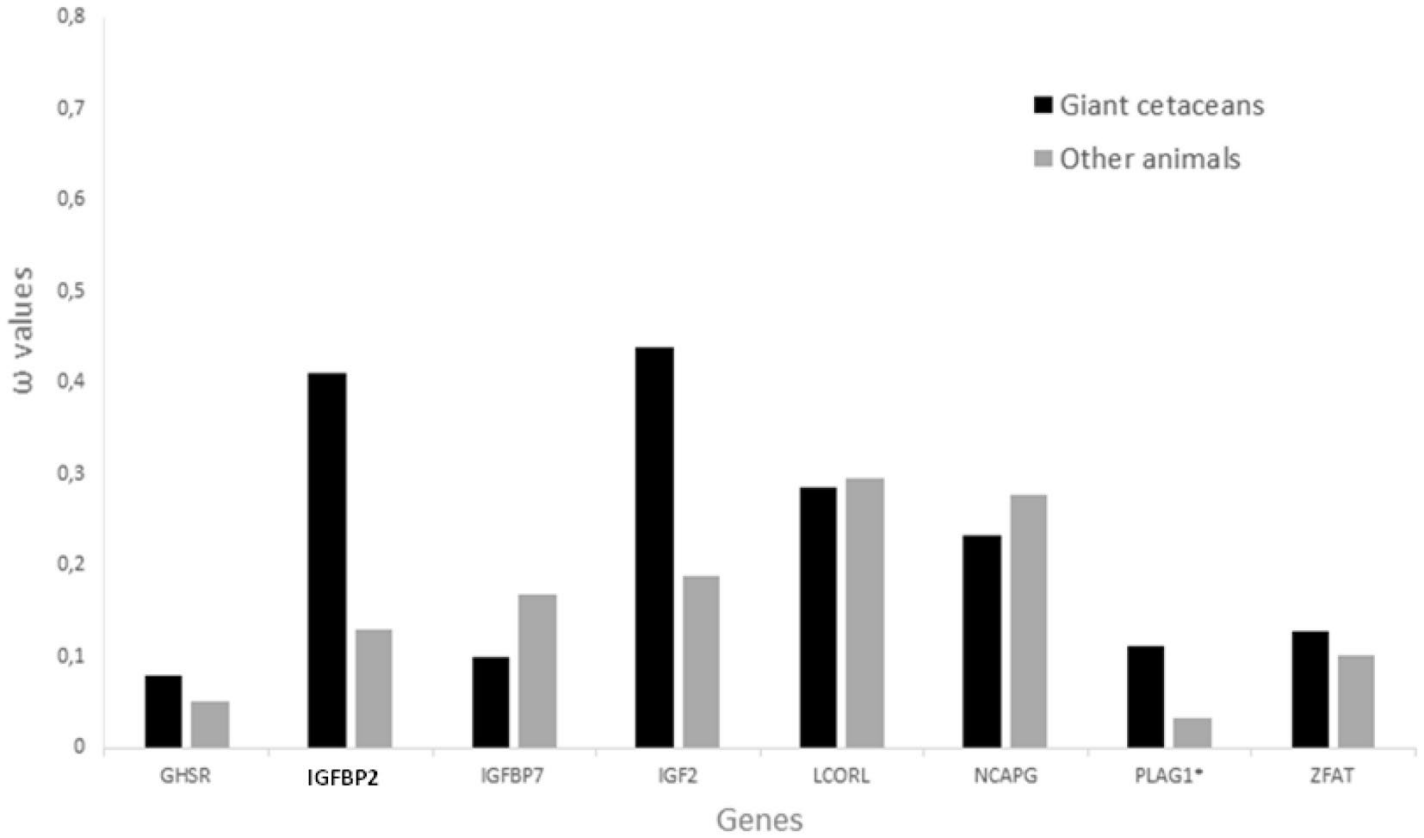
Comparison of ω values calculated using the two-ratio model in codeML for genes related to body size in giant cetaceans and other mammals. The two-model was better suited to the data (p < 0.05) in the PLAG1 gene, marked with an asterisk. The IGFBP2 gene, in bold, represents the statistically significant intensified selection that the RELAX program detected (*p* < 0.05) in giant cetaceans.

For RELAX analysis, the IGFBP2 gene was found to be under intensified selection (K > 1) using the first labeled scheme with the ancestral branch from mysticete lineage and sperm whale (Fig. 2), while no evidence of intensified selection was found using the second scheme, in which all giant cetaceans were labeled as a one group.

#### Branch-site models

To estimate selective pressures acting in specific sites on the giant cetacean lineages, we performed branch-site models using BUSTED, aBSREL, and codeML. BUSTED indicated the occurrence of positive selection in at least one site in at least one branch for the GHSR gene (p-value ≤ 0.05), aBSREL resulted in episodic positive selection in *Eschrichtius robustus* lineage for 0.34% of sites on the same gene, and CodeML found significant positive selection for site 211 for GHSR. CodeML also found positive selection for sites 134 and 353 for the NCAPG gene and the site 278 for IGFBP7. No significant positive selection was detected for the IGF2, LCORL, PLAG1, and ZFAT genes.

#### Site models

To find sites under positive selection within giant cetaceans, we used SLAC, MEME, and FUBAR (Table 1). SLAC resulted in some codons with ω values greater than 1 but no statistically significant signatures of positive selection. MEME found episodic selection/diversifying selection for sites 243 and 249 for GHSR, and 241, 466, and 885 in the NCAPG gene. FUBAR detected thirteen sites under episodic selection/diversifying selection for NCAPG (348, 373, 464, 466, 630, 885, 906, 908, 925, 940, 953, 969, and 991), and the 211 codon was identified as being under selection for GHSR gene.

**Table 1:** Codon positions under positive selection detected by the site model using FUBAR and MEME using genes trees.

Gene	Site model		Branch-site model	
	FUBAR	MEME	codeML	aBSREL
GHSR	211 (0.93)	243(0.03); 249(0.04)	211 (0.984)	0;34%
IGF2	0	0	0	0
IGFBP2	0	0	0	0
IGFBP7	0	0	278 (0.974)	
EGF	0	0	0	0
LCORL	0	0	0	0
PLAG1	0	0	0	0
ZFAT	0	0	0	0
NCPAG	348 (0.91), 373 (0.95); 464 (0.95), 466 (0.94), 630 (0.97); 885 (0.93); 906 (0.95); 908 (0.94); 925 (0.92); 940 (0.93); 953 (0.94); 969 (0.92); 991 (0.93)	241 (0.03); 466 (0.03); 885 (0.04)	134 (0.953); 353 (0.950)	0

Significance was assessed by Posterior Probability (PP) > 90% in FUBAR and *p* value < 0.05 in MEME. Detection of the position of codons under positive selection was carried out through the branch-site model using codeML and aBSREL. Significance is obtained through BEB (Posterior Probability (PP) > 0.90) for codeML and *p*-value < 0.05 in aBSREL.

**Figure 3:**
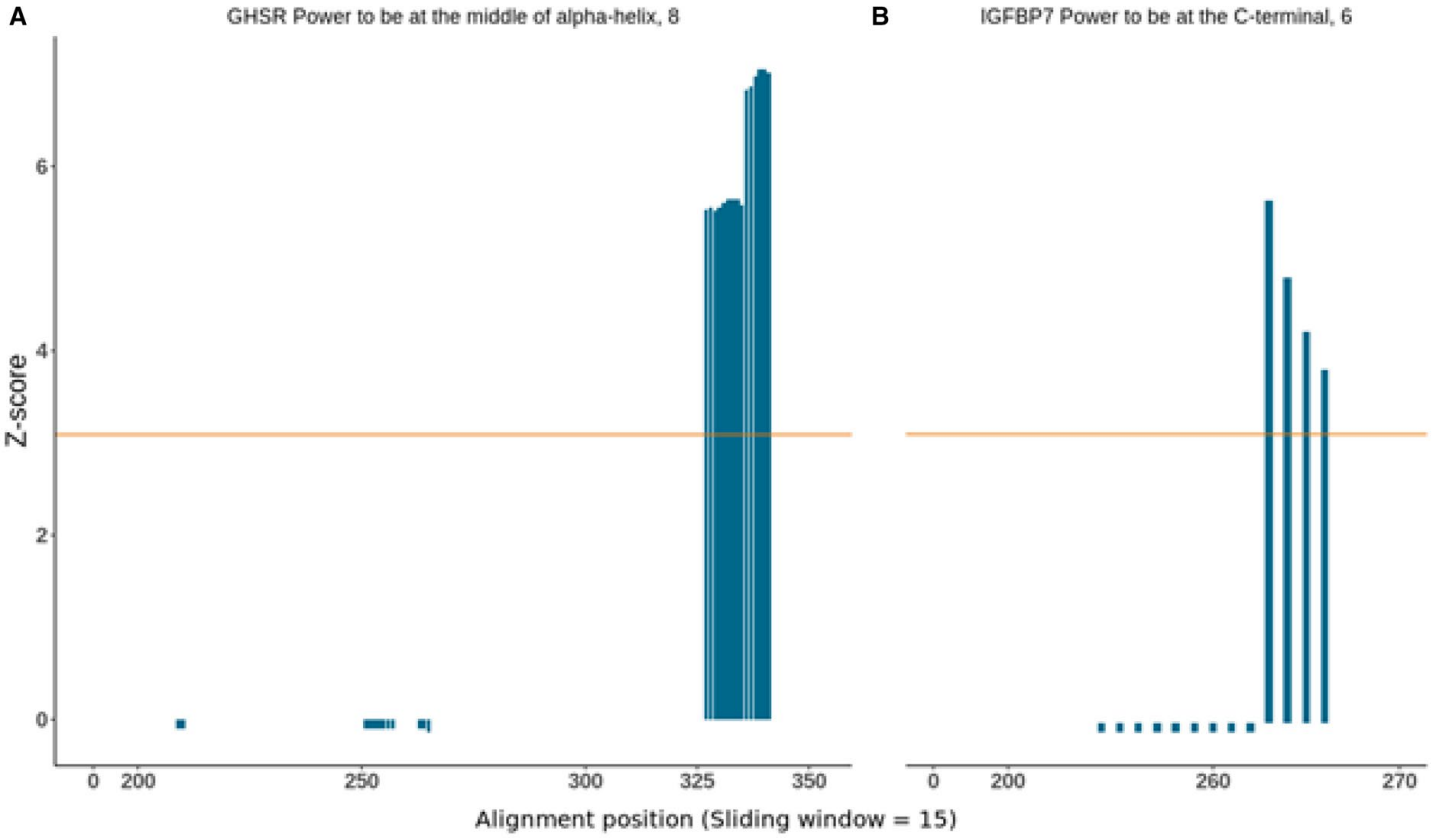
Detection of significant physicochemical amino acid changes using TreeSAAP and genes trees. This analysis was performed on the genes that presented higher ω values identified by codeML analysis in giant cetaceans. Only GHSR (**A**) and IGFBP7 (**B**) showed significant results. A highly significant z-score (z > 3.09, *p* < 0.01), represented here by the regions above the orange line, indicates more non-synonymous substitutions than assumed under the neutral model and therefore are interpreted as a result of positive selection. Respective property and category are shown above the graphs.

We found evidence for positive selection in the *power to be at the middle of alpha-helix* physicochemical property using TreeSAAP, with global z-scores > 3.09 (*p* < 0.001) for the GHSR gene, and in the *power to be at the C-terminal* physicochemical property for the IGFBP7 gene (Fig. 3).

## Discussion

This study presents the molecular evolution of genes related to body size in mammals, focusing on giant cetaceans. We found molecular signs of selection in the GHSR, IGFBP7, PLAG1, and NCAPG genes from the nine genes included in our dataset, with results converging with different algorithms. Furthermore, in the EGF gene, the presence of stop codons resulted in the interruption of the reading frame in all Mysticeti cetacean species, which is unique to this group.

The presence of stop codons in the epidermal growth factor (EGF) gene is a pseudogenization indicator. The stop codons start at position 948 (exon 3) in the alignment, and remain present until the last exon in different and multiple sites for all species of the Mysticeti group. Some stop codons likely share the same locus between particular mysticete species, but because the reading frame is very corrupted, the alignment is unreliable only in the group of toothless cetaceans, and we cannot state the site of the first mutation in stem mysticete lineage that led to the EGF pseudogenization in this group. To our knowledge, the inactivation of EGF in mysticetes is reported for the first time in this paper. The inactivation of protein-coding genes has already been associated with several traits of cetaceans^32^, such as vision^33^, loss of taste receptors^34^, hair loss^35^, and teeth in Mysticeti^36^, among others. From an evolutionary perspective and considering the occupation of the aquatic environment, the loss of function of these genes can be understood as part of the adaptation process and not only because of the relaxation of selection^37^. EGF binds to the epidermal growth factor receptor (EGFR), which then dimerizes or forms ErbB-2, ErbB-3, or ErbB-4 homologs, increasing the intracellular activity of tyrosine kinase, activating effects such as cell proliferation, apoptosis and angiogenesis, embryonic growth, and tissue regeneration^38^. In addition, EGF has been associated with the development and eruption of teeth, being found within the dental follicle, in the alveolar bone related to ameloblasts (the cells that form tooth enamel), and during the pre-functional stage of tooth eruption in rats, animals in which EGF injections in neonates significantly stimulated the eruption of the incisor teeth^39–41^.

Teeth loss occurred in the common ancestor of all extant mysticete cetaceans, and ontogenetic evidence suggests that teeth develop rudimentarily in fetuses in this group; however, they are later aborted and reabsorbed before enamel formation^42,43^. Thus, evidence of pseudogenization of the EGF gene only in Mysticeti is likely related to the loss of teeth and the appearance of baleen. The baleen, for this group, was an evolutionary innovation that allowed whales to exploit a new foraging niche: filtration, which was previously identified as a probable trigger for gigantism^26,44^. Furthermore, the loss of EGF functionality and its role in important components of homeostasis, such as the kidneys, would be compensated for by the role of other genes in the EGF family and other pathways, an overlap of functions that has been reported in rats that did not have active EGF and yet had a normal and healthy phenotype^44^.

Cetacean body size seems to respond to intense selective pressures imposed by the aquatic environment. Factors such as thermoregulation, feeding ecology, and space availability shaped the gigantic body proportions of these animals^14–16^. Furthermore, migratory behavior, like the one performed by the blue whale that transits in polar waters, affects body size following Bergmann's rule, which states that animals living in colder climates are generally larger than those living in warmer regions^45^. The combination of these external factors can now be studied at the molecular level due to advances in genomic technologies. For example, a recent study reported signs of positive selection in body size genes in cetaceans: in small species, the genes under selection related to short size were ACAN, OBSL1, and GRB10; in the large cetaceans, the selection was identified in the CBS, EIF2AK3 and PLOD1 genes, all related to the large size^46^. Together, their results and the results from this study aid to our understanding of the evolutionary panorama of large body evolution, which is a complex feature that affects many genetic pathways.

Our results identified the GHSR with evidence for positive selection in the physicochemical property *Power to be at the middle of alpha-helix,* with global z-scores > 3.09 (*p* < 0.001) using TreeSAAP. BUSTED found evidence of positive selection for the GHSR gene (p-value ≤ 0.05) and aBSREL in the sperm whale for 0.32% of sites. Also, codons 243 and 259 were identified as being under positive selection by MEME, and 211 were identified by the codeML branch-site model (98%) and FUBAR (0.93 p.p). In this site, the sperm whale (*Physeter catodon*) was the only one to present glycine (G). This nonpolar amino acid that is compatible with hydrophilic and hydrophobic environments, while all other animals had threonine (T), which is polar and highly soluble in water. This modification is important because glycine is considered a "helix breaker" once it disrupts the regularity of the α helical backbone conformation since it lacks a β carbon, which is associated with more conformational freedom than other residues^47,48^. As mentioned before, the results reinforce that large phenotypes may evolve by different paths. It is worth noting that changes in specific species, such as the different site in the sperm whale—the only odontocete classified as a giant, may be related to characteristics of that species, involving the large body size or other characteristics affected by the gene. GHSR is an endogenous ligand that can stimulate, through its ghrelin ligand, the release of growth hormone through the pituitary gland and thus increase appetite, regulate body weight, energy metabolism, and fat accumulation^49^. In addition, it is associated with the secretion of gastric acid, control of cell proliferation, apoptosis, lactation, and cardiovascular pressure^50–52^. This gene has been linked to increased body size in cattle, sheep, and pigs^30,53–55^. In most cases, the increase in body size in these animals results from changes in a few sites, but these changes were not found in giant cetaceans. Nevertheless, this may indicate that minor changes in this gene can result in phenotypic modifications.

IGFBP7 is a 27 kD protein and a member of the IGFBP superfamily, responsible for the viability of insulin-like growth factors (IGFs)—molecules involved in promoting cell growth and division^56^. Evidence suggests that IGFBP7 acts as an oncosuppressor gene in prostate, breast, lung, and colorectal cancer due to its regulatory action related to cell proliferation, cell adhesion, cell senescence, and angiogenesis^57,58^. This repressor activity may arise with the interruption of the cell cycle in the G1 phase, induction of senescence, and an increase in the level of cell death through apoptotic cells^59^. In addition, it has already been observed that the higher the body mass index, the greater the expression of IGFBP7. This is possibly associated with the fact that obesity is an agent related to senescence, and IGFBP7 is secreted by senescent cells. This relationship that may indicate a compensation mechanism for organisms that reach high body mass^60^. In our analyses, we found evidence for positive selection in the physicochemical property *power to be at the C-terminal* with global z-scores > 3.09 (*p* < 0.001) using TreeSAAP. Furthermore, the branch-site implemented in codeML inferred that site 278 is under positive selection. In this site, the blue whale (*Balaenoptera musculus*) shows a loss of codons and no expressed amino acids. In contrast, the gray whale (*Eschrichtius robustus*) presents the nonpolar and hydrophobic methionine (M), while the other animals had glutamate (E), a polar amino acid. In summary, it seems that IGFBP7 is related to two main characteristics of giant cetaceans: increase in body size and suppression of cancer. Some cancer suppressor genes have already been reported to be under positive selection for cetaceans^61^, and IGFBP7 is likely to be one more.

NCAPG (Non-SMC Condensin I Complex Subunit G) is a gene strongly associated with increased body size and weight gain. It has been reported to be linked to birth weight, withers height, feeding efficiency, and pubertal growth in bovine species^62–64^. Besides cattle, this gene has been linked to growth in horses, donkeys (*Equus asinus*), pigs, humans, and chickens^65–71^. NCAPG also presented many sites evolving under positive selection, thirteen in the FUBAR program (348, 373, 464, 466, 630, 885, 906, 908, 925, 940, 953, 969, and 991), three in MEME (241, 466, and 885) and two in the codeML branch-site model (134 and 353) with some of them recovered by different methods, such as the 466, and 885 identified by FUBAR and MEME. Accordingly, this gene is probably the one that may be most directly involved in cetacean gigantism from our dataset, acting directly on two important characteristics—growth and weight gain.

In the same direction, PLAG1 (Pleomorphic Adenoma Gene 1) is a gene associated with growth in cattle^72^, pigs^73^, and sheep^74^, mainly in traits such as height, knuckle, biceps, and shank^75^. This gene encodes a zinc finger protein family, a nuclear protein transcription regulator^76^, playing an important role in the transcriptional regulation of growth factors such as IGF2, which is related to embryo growth and cell survival^77^. This is a candidate gene for future analysis with new parameters since it is related to growth in several animals, and mutations have been described as promoting changes in height^78^. In our analyses, PLAG1 was the only gene with evidence of positive selection by the codeML branch model.

Collectively, our results indicate four genes likely to be involved in increasing body size in giant cetaceans. Some of these genes, such as GHSR and IGFBP7, may also be responsible for mitigating the possible consequences of extreme size, as they control important aspects of the cell cycle. Hypothetically, being a giant has severe consequences, such as increased chances of developing cancer, in addition, cetaceans are long-lived animals, which is also related to this disease. Giant cetacean species (larger than 10 m) included in this study live longer than 30 years, with the humpback whale (*Megaptera novaeangliae*) reaching 50 years, and the blue whale (*Balaenoptera musculus*) and the fin whale (*Balaenoptera physalus*) reaching up to 90 years, while the bowhead whale (*Balaena mysticetus*) is the longest lived mammal known, with a lifespan of 200 years^79^. Despite these triggers, giant animals are less likely to develop cancer than small animals, a logical contradiction called Peto's Paradox that suggests the existence of a mitigation mechanism^7^. Thus, genes that act on body growth and control of the negative aspects mentioned above through cell control, cell division, and tumor suppression could be targets of natural selection, allowing these animals to become giants, live longer, and have great body mass.

It is worth remembering that body size is a complex characteristic that involves many factors and molecular pathways. Throughout cetacean evolutionary history, different lineages had different selective pressures on different genes that could result in the same large body phenotype. This could be the case for the sperm whale, an odontocete as large as a mysticete. Interestingly, genes previously reported as associated with large sizes in artiodactyls, such as LCORL and ZFAT, apparently do not show the same effect in cetaceans, highlighting how large sizes may arise from different pathways from different genes in different lineages.

## Conclusion

In summary, here we investigated the molecular evolution of genes possibly related to increased body size in giant cetaceans. We found evidence for positive selection at the coding level for sites in the GHSR, IGFBP7, PLAG1, and NCAPG genes. Besides that, we found evidence of pseudogenization of the EGF gene in the Mysticeti lineage, an event likely related to teeth loss in these cetaceans, which could be connected with the emergence of the baleen plate filter system. In conclusion, our study provides new perspectives on the evolution of cetacean gigantism, reinforcing the selective pressures of the aquatic environment, the various possibilities of action of natural selection on different genes that have similar functions depending on specific characteristics for each species, and indicating that pseudogenization is also an adaptive process for this group.

## Material and methods

### Sample data

We focused on the genes GHSR, IGF2, IGFBP2, IGFBP7, and EGF from the growth hormone/insulin-like growth factor axis and the genes NCAPG, LCORL, PLAG1, and ZFAT that are associated with increased body size in artiodactyls. The cetacean group was composed of 12 odontocetes (*Lagenorhynchus obliquidens*, *Neophocaena asiaeorientalis*, *Delphinapterus leucas*, *Tursiops truncatus*, *Orcinus orca*, *Monodon monoceros*, *Globicephala melas*, *Lipotes vexillifer*, *Physeter catodon*, *Phocoena sinus*, *Sotalia fluviatilis,* and *Sotalia guianensis*) and seven mysticetes (*Balaenoptera acutorostrata*, *Balaena mysticetus*, *Eschrichtius robustus*, *Eubalaena japonica*, *Megaptera novaeangliae*, *Balaenoptera physalus*, and *Balaenoptera musculus)*, totaling 19 species*.* The coding sequences for the species *Balaena mysticetus* came from the public platform *The Bowhead Whale Genome Resource*. In addition, *Sotalia fluviatilis* and *Sotalia guianensis* were sequenced by our laboratory (data not published). All other coding sequences were retrieved from GenBank and the accession numbers can be found in the Table 1. The sequences were retrieved according to their availability in the databases and quality. Thus, the dataset is not the same for all genes, but at least one giant cetacean is present for all of them. The sequences were aligned using the MUSCLE algorithm^80^ and we used Geneious software v. 7.1.3^81^ to remove fragmented sequences that were larger or smaller than expected.

True gigantism in cetaceans is defined as body length above 10 m^82^. According to this criterion, the species classified as giants were *Physeter catodon*, *Balaena mysticetus*, *Eschrichtius robustus*, *Megaptera novaeangliae*, *Eubalaena japonica*, *Balaenoptera physalus*, and *Balaenoptera musculus* (Table 2).

**Table 2:** Average size in meters of all cetacean species included in this study.

Infraorder	Species	Size (m)
Odontocetes	*Phocoena sinus*	1.4
	*Sotalia fluviatilis*	1.5
	*Lipotes vexillifer*	2.5
	*Neophocaena asiaeorientalis*	2.0
	*Sotalia guianensis*	2.2
	*Lagenorhynchus obliquidens*	2.2
	*Tursiops truncatus*	3.8
	*Delphinapterus leucas*	4.2
	*Monodon monoceros*	5.0
	*Globicephala melas*	5.7
	*Orcinus orca*	8.0
	***Physeter catodon***	**20.0**
Mysticetes	*Balaenoptera acutorostrata scammoni*	8.5
	***Eschrichtius robustus***	**15.0**
	***Eubalaena japonica***	**18.0**
	***Balaena mysticetus***	**17.0**
	***Megaptera novaeangliae***	**19.0**
	***Balaenoptera physalus***	**25.0**
	***Balaenoptera musculus***	**30.0**

Significant values are in [bold].

True gigantism in cetaceans was defined as body length above 10 m. *Physeter catodon*, *Balaena mysticetus*, *Eschrichtius robustus*, *Megaptera novaeangliae*, *Eubalaena japonica*, *Balaenoptera physalus*, and *Balaenoptera musculus* are classified as giants. Information from Encyclopedia of Marine Mammals.

### Molecular evolutionary analyses

To estimate the role of natural selection in our focus genes we estimated the value of ω (*dN*/*dS*), which is the ratio of the rate of non-synonymous substitutions (*dN*) to the rate of synonymous substitutions (*dS*), where ω < 1 indicates purifying selection, ω = 1 suggests neutral evolution, and ω > 1 indicates positive selection^83,84^. Different approaches were applied: the branch model that identifies how ω varies through the branches of the phylogeny, site-models that detect variations of ω in distinct sites, and the branch-site model that integrates both approaches^83–85^. The branch model and branch-site models were performed for a dataset with the species of interest and other mammals labeling the ancestral branches that resulted in these giant animals. In contrast, the site-models were performed only with the cetacean species classified as giants (Supplementary Figs. S1 and S2). Such tests were done on species trees, with relationships based on Beck and Baillie^86^ for mammals, while phylogenetic relationships among cetaceans are based on McGowen et al.^87^.

#### Branch models

To check whether the value of ω for giant cetaceans was different compared to other animals of the phylogenetic tree we used a branch model available at codeML within the PAML package^85^ that allows the variation of ω in the branches of the phylogeny. First, we used the one-ratio model that estimates a single value of ω for all branches. Then, the free-ratio model was applied, calculating ω for each branch. Finally, we used the two-ratio model, where we inferred a value of ω for giant cetaceans and another for the rest of the phylogeny. In this case, the interest group was identified as a foreground branch, while the algorithm treated the other unmarked ones as background branches^88^. We tested two scenarios, first labeling the ancestral branches that led to the stem mysticete lineage and the branch of the sperm whale (*Physeter catodon*), and second labeling all cetaceans' species with body size larger than 10 m classified as giants as one group to compare within non-giants cetaceans. The same configuration was used in RELAX, a method to test whether the selection was relaxed (K < 1) or intensified (K > 1) on a portion of branches specified a priori in the phylogeny^89^.

#### Site models

For site model analyses, the dataset comprised only the sequences of giant cetaceans. FUBAR software (Fast Unconstrained Bayesian AppRoximation) was used to identify sites that may have experienced generalized diversification or purifying selection by estimating the ratios between *dN* and *dS* substitution rates for each site where posterior probability (PP) of ω > 1 is greater than 95%^90^. SLAC (Single-Likelihood Ancestor Counting) was also used. This algorithm combines maximum-likelihood (ML) and counting approaches to calculate the ratios between *dN* and *dS* rates by site given a codon alignment^91^. Finally, MEME (Mixed-Effects Model of Evolution) looked for evidence of episodic or diversifying selection at individual sites allowing ω to change from site to site and branch to branch^92^.

TreeSAAP v.3.2^93^ relies on the MM01 model implemented in baseML from the PAML package^85^ using phylogeny to reconstruct the most likely ancestral states for the gene sequences, detecting selection at the amino acid level. The software assigns weight values to the non-synonymous codon changes, for which overall physicochemical effects are assessed using a model with 31 physicochemical amino acid properties, with these changes ranging from 1 (conservative) to 8 (radical change). Positive selection is checked through a z-score to calculate deviation from neutral evolution. A highly significant z-score (z > 3.09, *p* < 0.01) indicates more non-synonymous substitutions than assumed under the neutral model, and only amino acid changes with a score between 6 and 8 and with a positive z-score < 0.001 were considered^94^.

#### Branch-site models

The branch-site model was used to identify whether some sites were subjected to the action of positive selection in the group of giant cetaceans. For this analysis in codeML, the interest group (i.e., all giant cetaceans) was labeled in the phylogeny as *foreground branches*, where sites with ω > 1 are allowed, and the rest of the tree was labeled as *background branches*, where sites with ω > 1 are not allowed. Model A was then used against the null model.

Two other branch-site tests were performed, from the HyPhy package on the DataMonkey portal^91,95^. BUSTED (Branch-Site Unrestricted Statistical Test for Episodic Diversification), which identifies a gene that experienced positive selection in at least one site in at least one branch^96^, and aBSREL (Adaptive Branch-Site Random Effects Likelihood), which allows positive selection in unspecified branches of the tree. To avoid excessive parameterization, aBSREL uses the Akaike Information Criterion correction (AICc) to estimate the ideal number of categories per branch instead of defining that each branch must be equipped with three classes. In addition, the Bonferroni-Holm approach was used to control false-positive rates^84,97^.

## Supplementary Information

Supplementary Figure S1.

Supplementary Figure S2.

## Data Availability

The datasets analysed during the current study are available in the Supplementary Files.
