# Supplementary material for: The molecular evolution of genes previously associated with large sizes reveals possible pathways to cetacean gigantism
Source: Sci Rep. 2023 Jan 19;13:67. doi: 10.1038/s41598-022-24529-3 (PMC9852289; doi:10.1038/s41598-022-24529-3)
Supplement: Supplementary file 1 — Supplementary Figure S1. [file 41598_2022_24529_MOESM1_ESM.pdf]

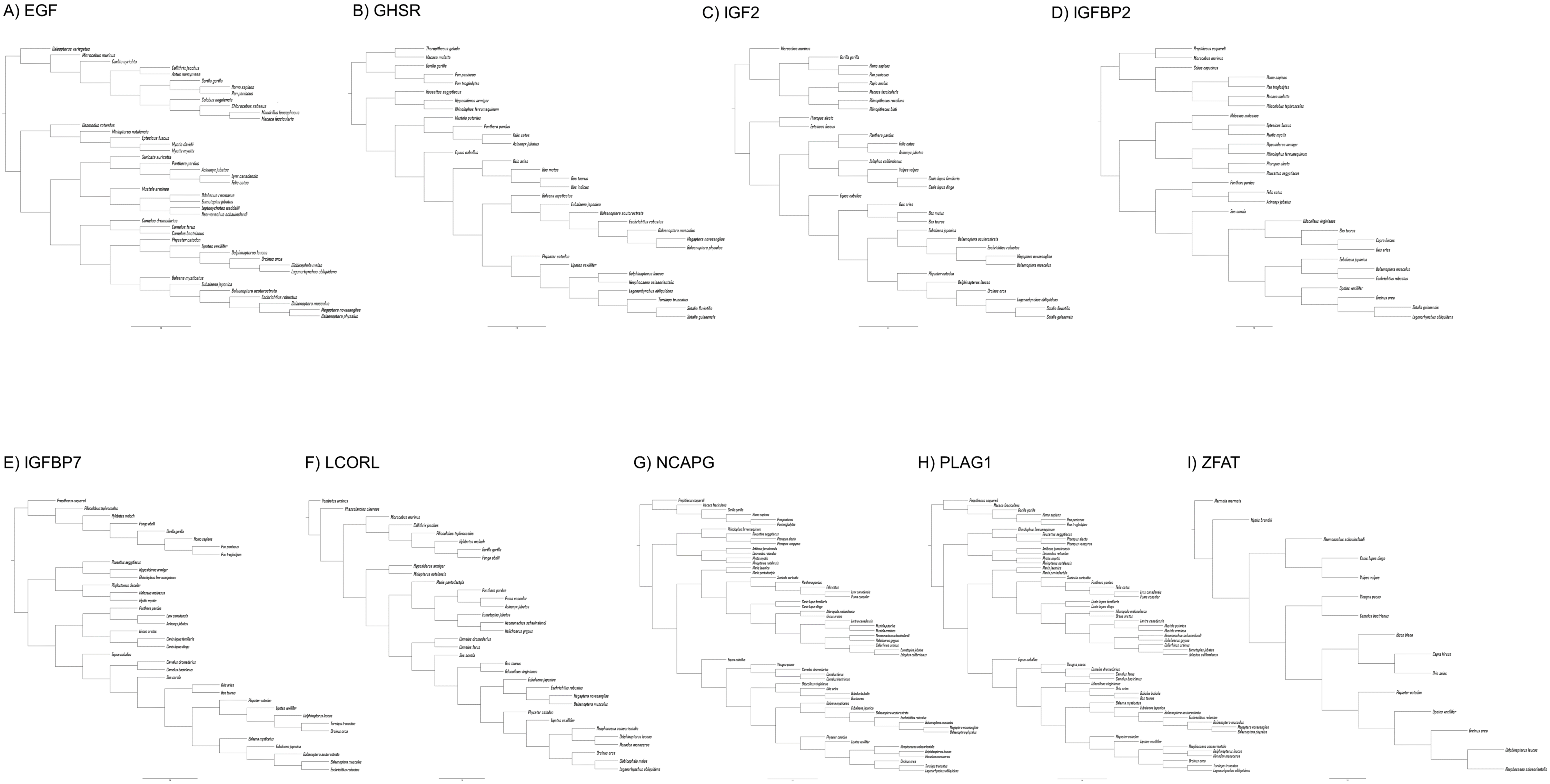

Figure S1: Species trees by each gene using dataset I, in which there are cetaceans and other groups of mammals.
