# Supplementary material for: The molecular evolution of genes previously associated with large sizes reveals possible pathways to cetacean gigantism
Source: Sci Rep. 2023 Jan 19;13:67. doi: 10.1038/s41598-022-24529-3 (PMC9852289; doi:10.1038/s41598-022-24529-3)
Supplement: Supplementary file 2 — Supplementary Figure S2. [file 41598_2022_24529_MOESM2_ESM.pdf]

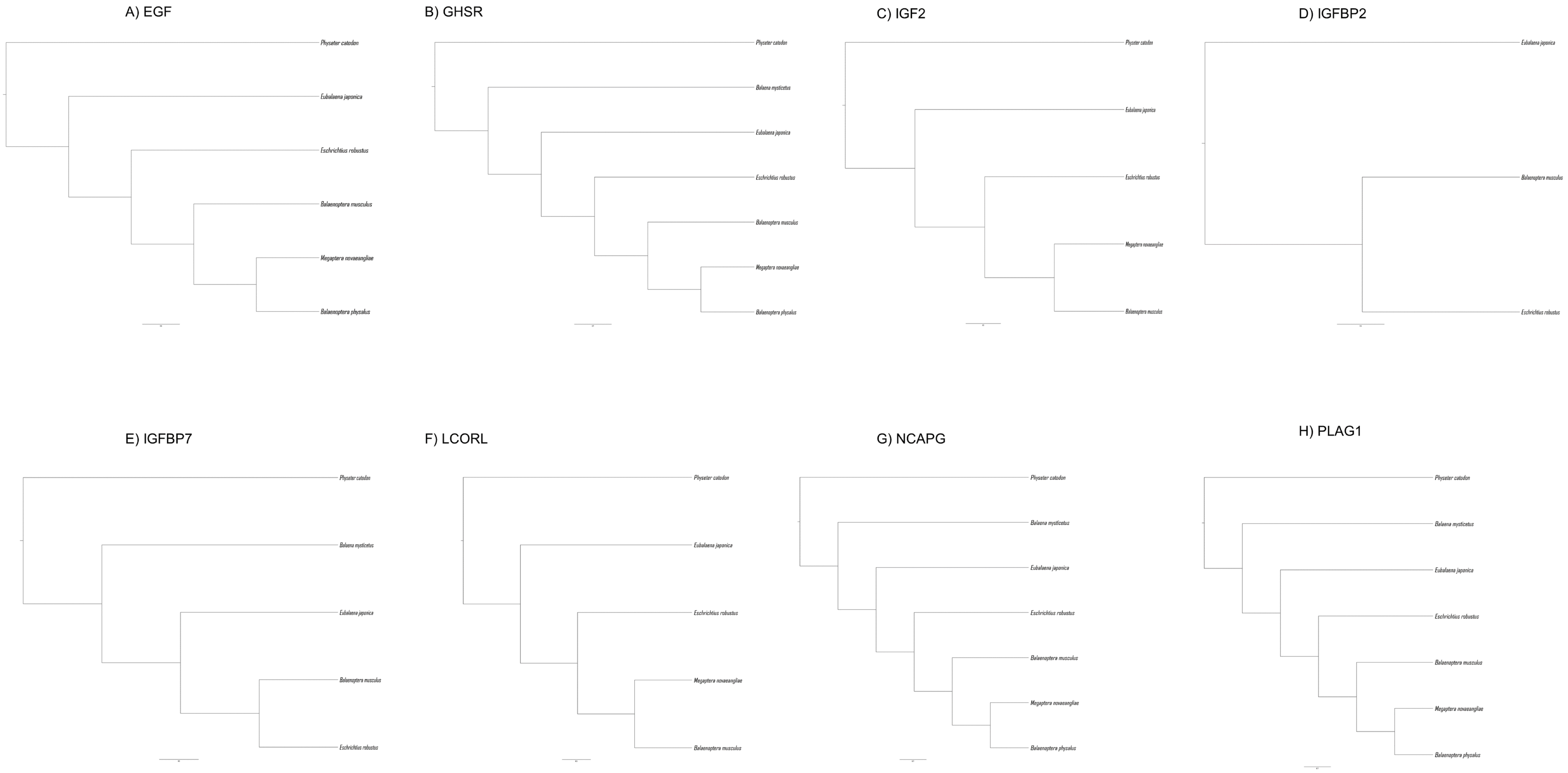

Figure S2: Species trees by each gene using dataset II, in which there are only giant cetaceans (larger than 10 meters).
